# Supplementary material for: Resolving the full spectrum of human genome variation using Linked-Reads
Source: Genome Res. 2019 Apr;29(4):635–45. doi: 10.1101/gr.234443.118 (PMC6442396; doi:10.1101/gr.234443.118)
Supplement: Supplemental Material [file supp_gr.234443.118_Supplemental_longranger-2.2.zip › longranger-2.2.tar.bz2!/lr2.2-public/lib/go/src/code.google.com/vitess/docs/resources/percona-2015-vitess-and-kubernetes.pdf]

YouTube Vitess

Scaling MySQL in the cloud with Vitess and Kubernetes

Percona Live MySQL Conference
April 14, 2015

Anthony Yeh, Software Engineer, YouTube

Vitess in a Nutshell

Vitess Overview

Performance

- Connection pooling
- Query deduplication
- Transaction manager
- Rowcache

Protection

- Query sanitation
- Query blacklist
- Query killer
- Table ACLs
- Health check

Monitoring / Analysis

- Performance analysis
- Query stream
- Update stream
- MapReduce (Hadoop)

Management

- Reparent (master failover)
- Web console
- Multi-region coordination

Sharding

- Range-based or app-defined
- Live resharding
- Split by row
- Split by table

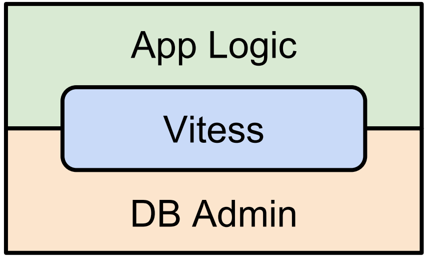

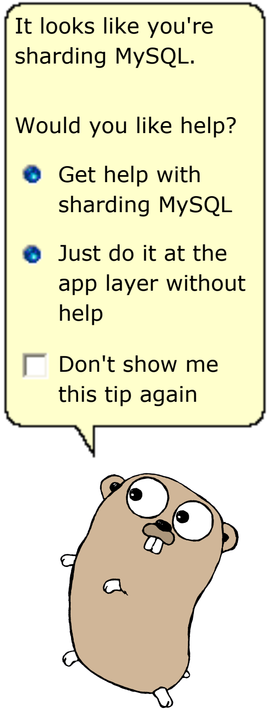

Details: vitess.io/overview

App View of Vitess

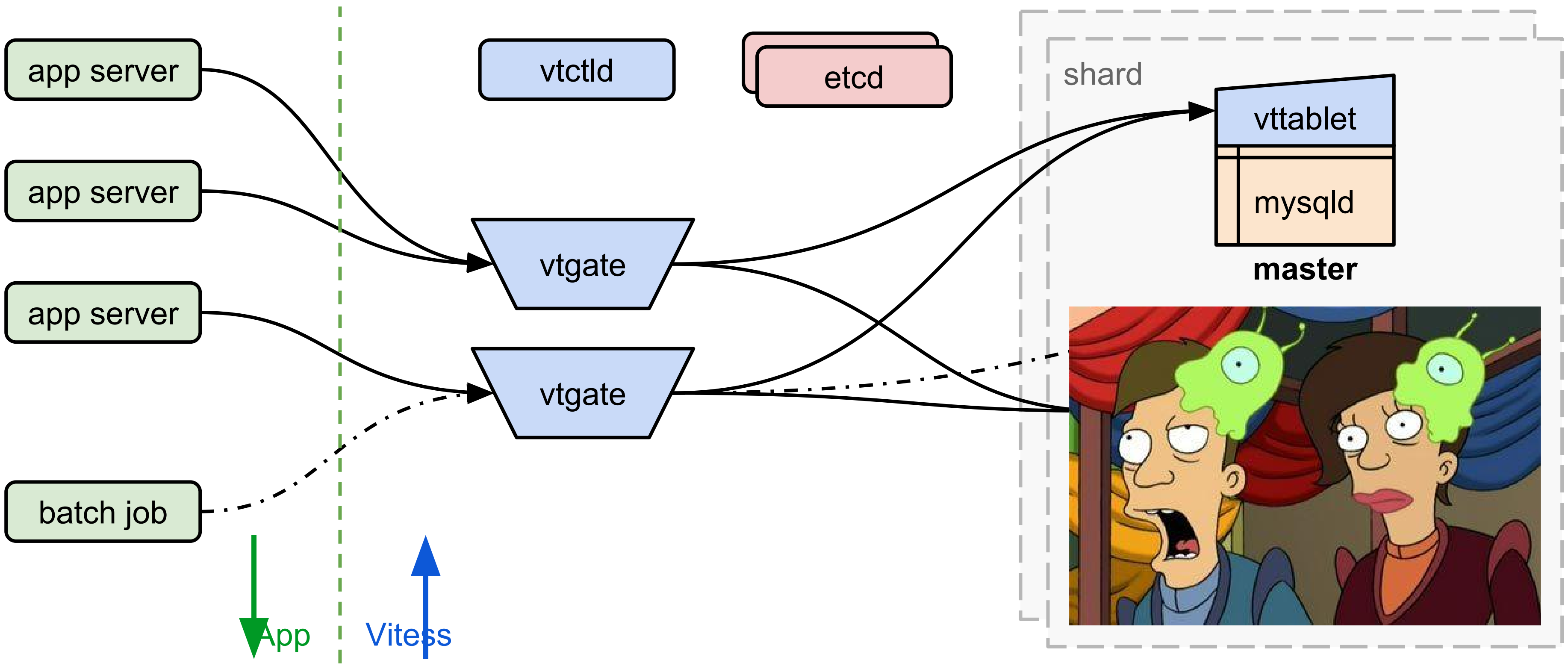

Admin View of Vitess

GUI / CLI / RPC

- Examine/modify tablets, shards, keyspaces
- Backup/Restore
- Reparent (failover)
- Schema changes
- Live migrations (e.g. resharding)

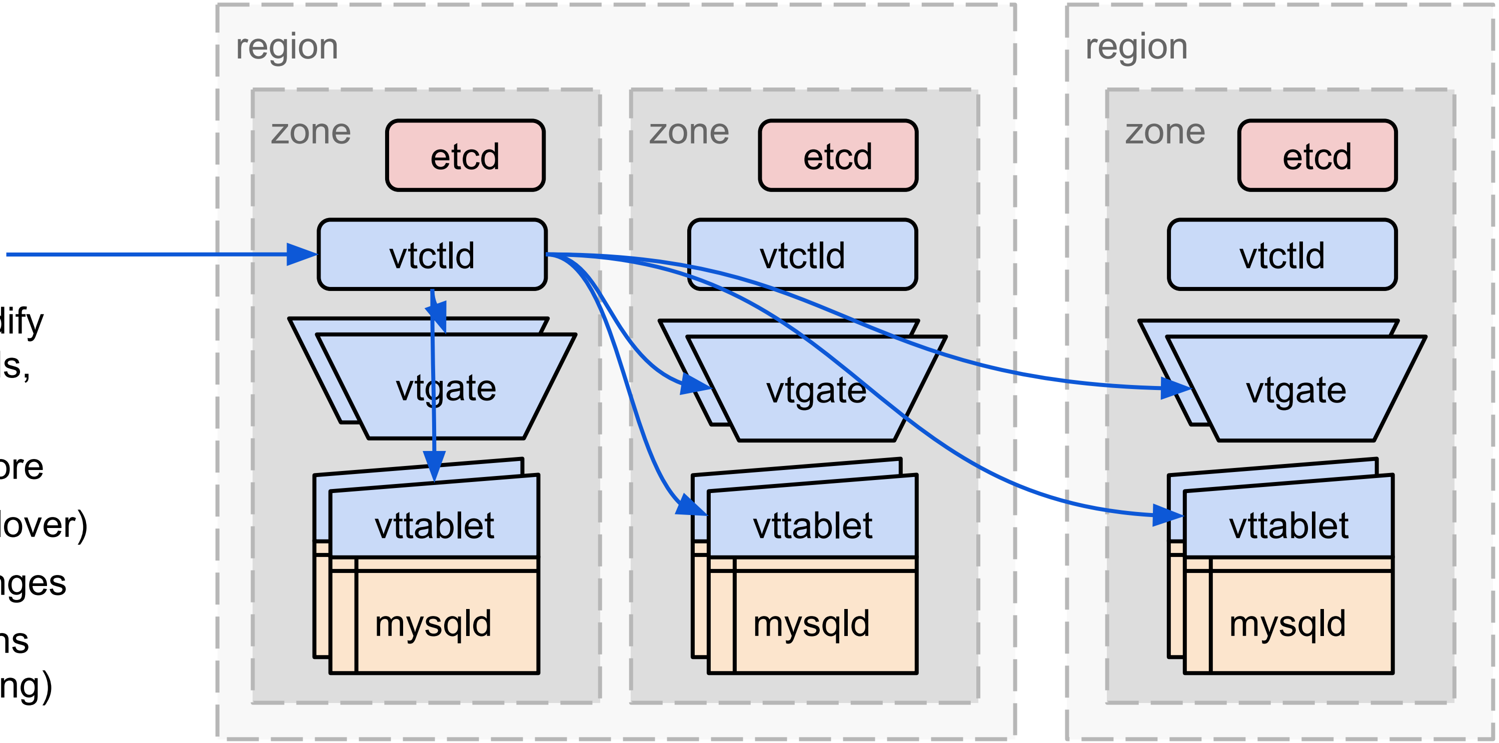

Performance and Protection

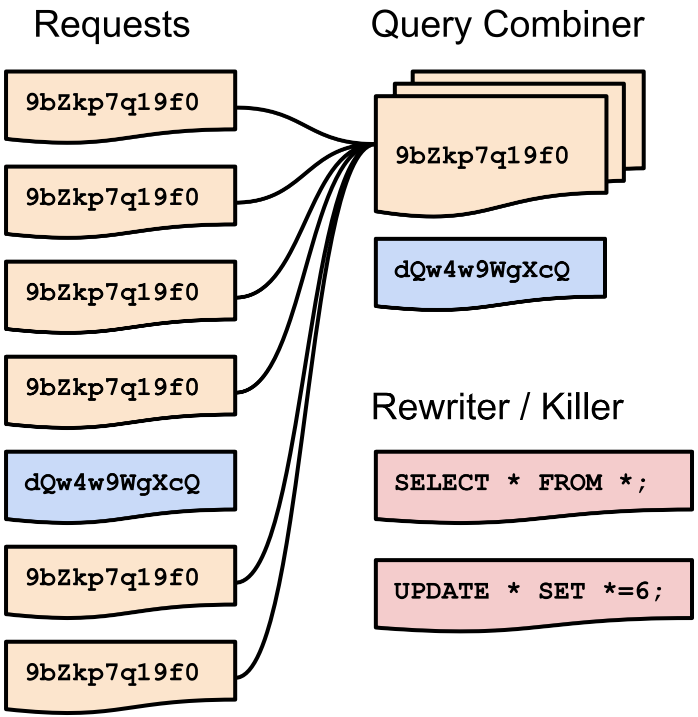

Row Cache (Vitess)

id	title	views	...
9bZkp7q19f0	강남스타일	2298169867	...

Block Cache (MySQL)

id	title	views	...
...	...	...	...
9bZkp7q19f0	강남스타일	2298169867	...
dQw4w9WgXcQ	...	114838229	...
...	...	...	...

Sharding Schemes

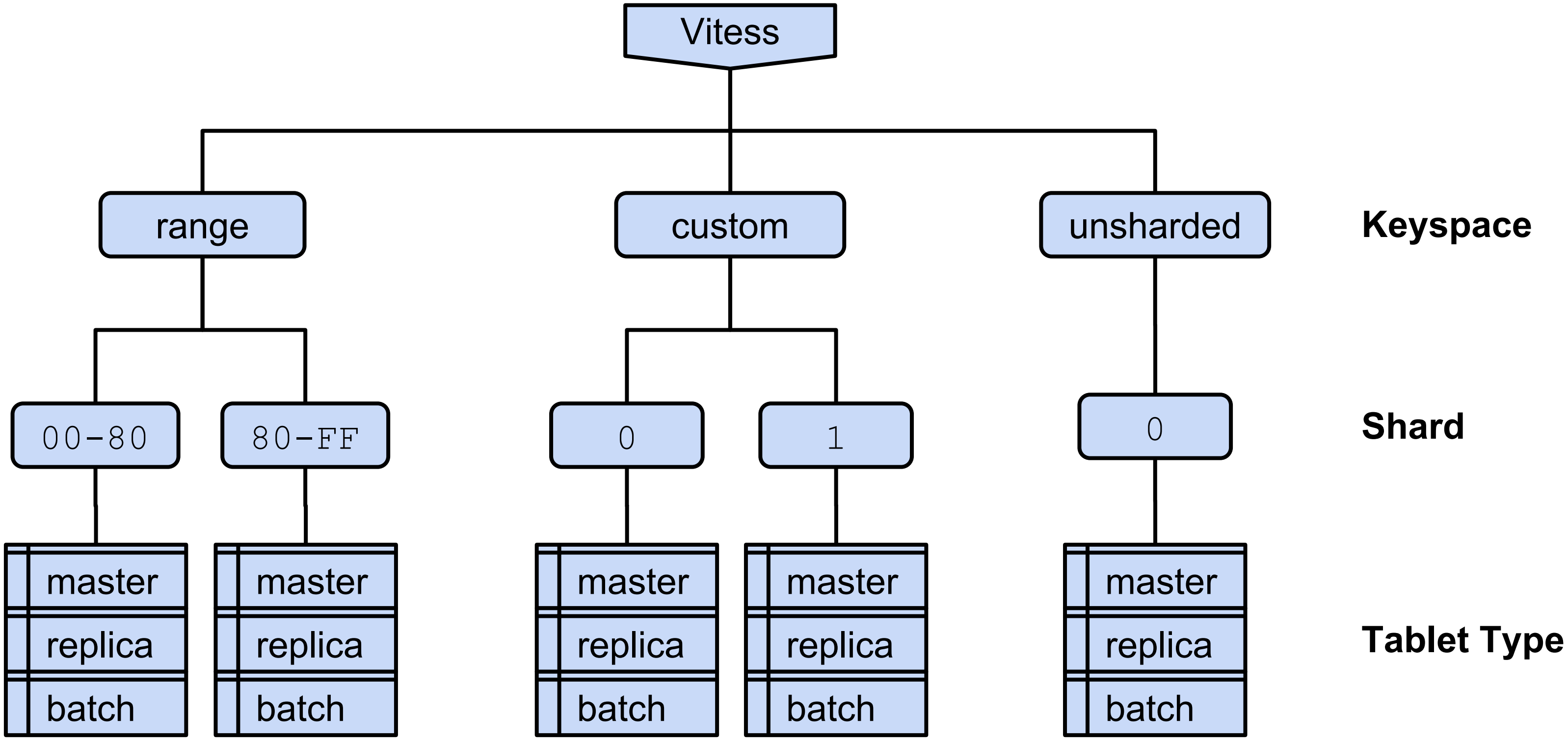

Vitess in a Container Cluster

Docker and Kubernetes

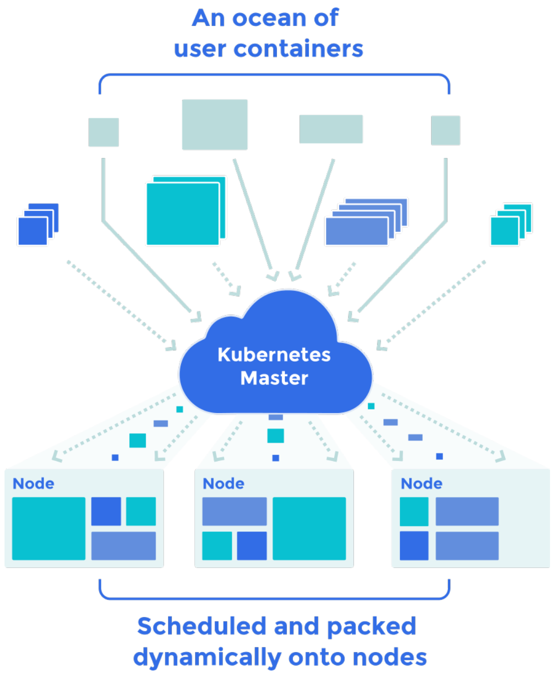

kubernetes.io

- **Container:** Lightweight kernel-level VM
- **Docker:** Container image compiler and runtime
- **Kubernetes:** Container Cluster OS

Shortcut for starting Vitess on Google Container Engine:

```
$ go get github.com/youtube/vitess/go/cmd/vtctlclient
$ cd $GOPATH/src/github.com/youtube/vitess/examples/kubernetes
$ export KUBECTL='gcloud alpha container kubectl'
$ ./cluster-up.sh
```

Detailed guide: vitess.io/getting-started

Vitess in Kubernetes

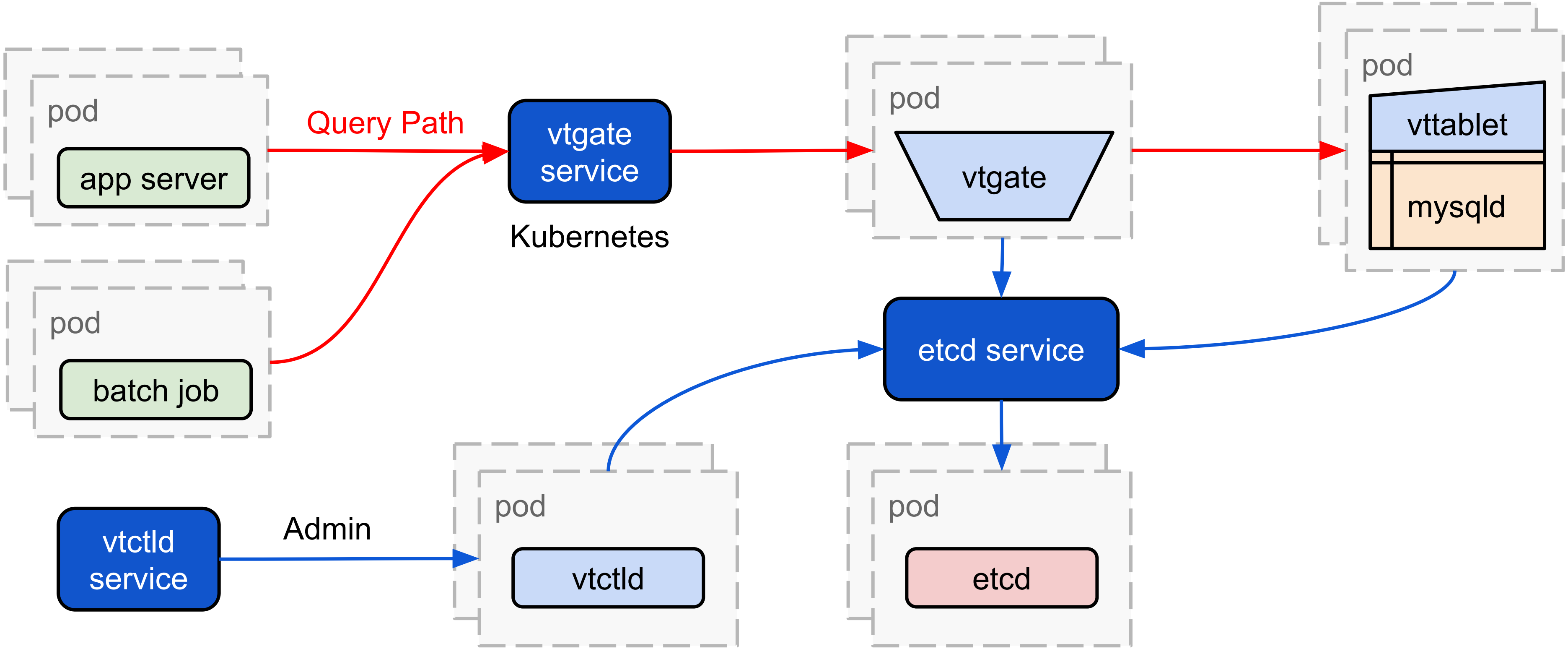

Vitess in the Cloud

Transparent Rerouting

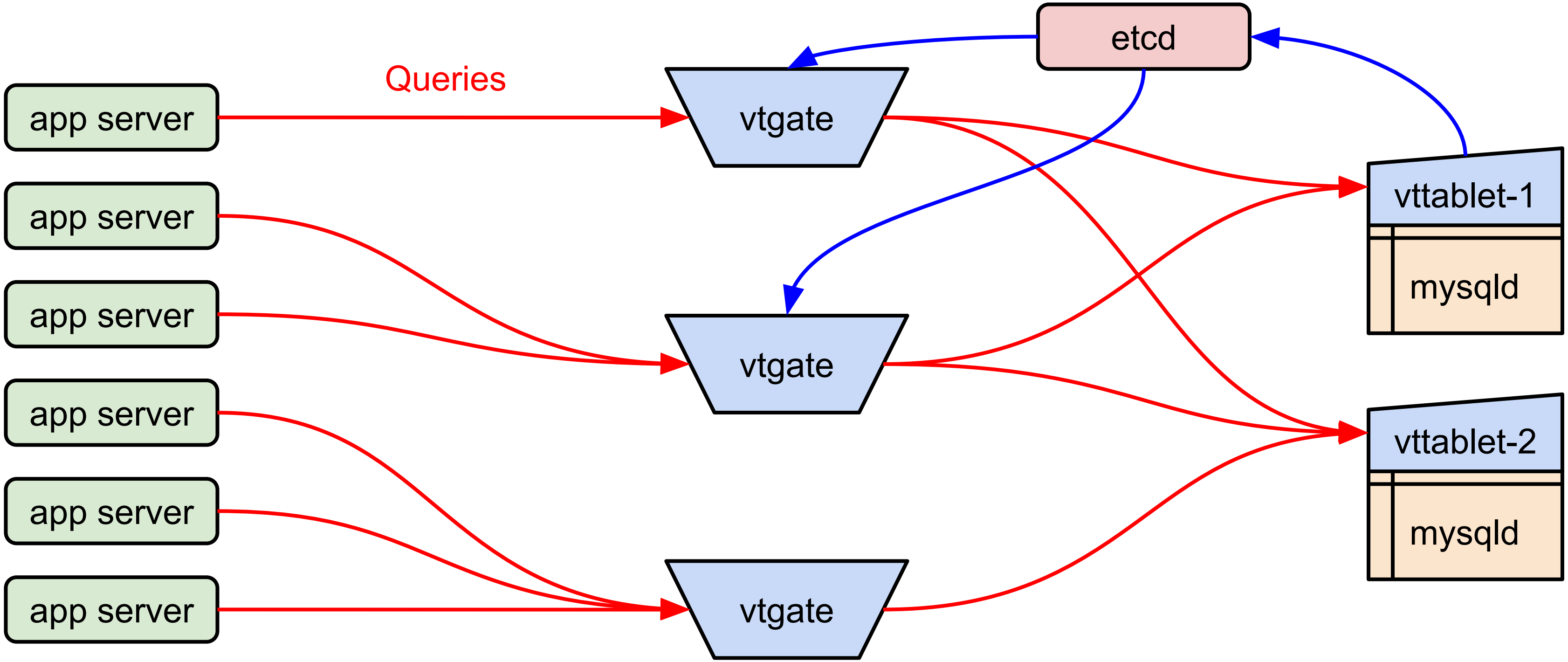

Routine Reparent

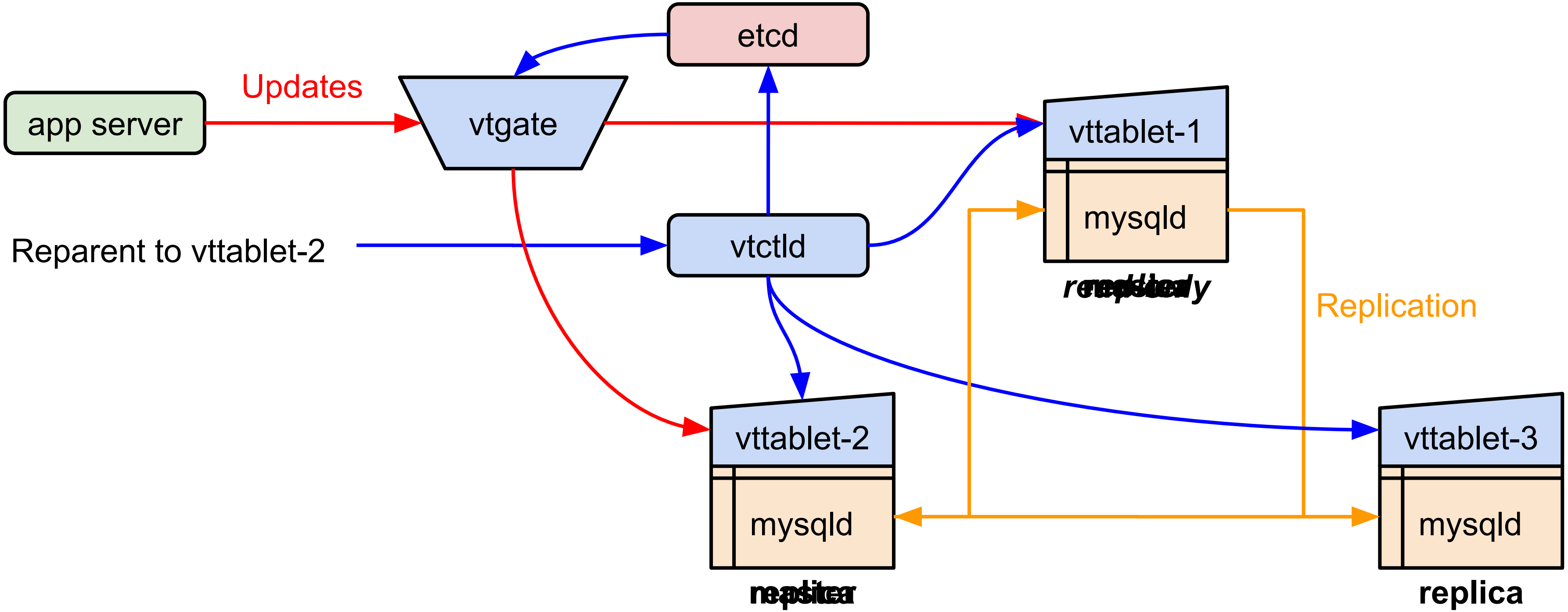

Live Resharding

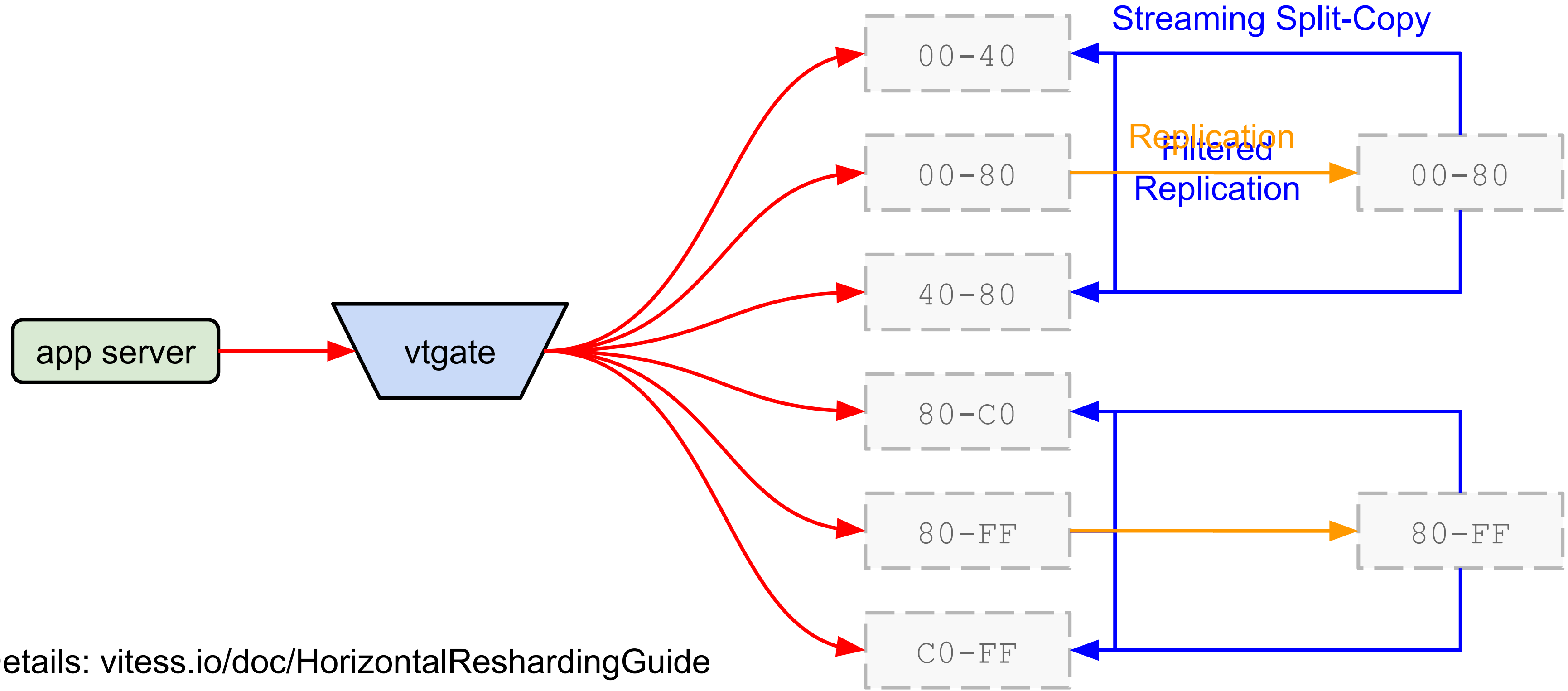

Details: vitess.io/doc/HorizontalReshardingGuide

Vitess in the Future

Roadmap

- MySQL 5.6
- Benchmarks (YCSB)
- VTGate V3
  - Automatic lookup tables
  - Cross-shard auto_increment
  - MySQL binary protocol
- High-level automation
  - One-click resharding
  - Rolling (OOB) schema changes
- More documentation
  - Architecture / design
  - How-to guides
- Kubernetes
  - API server proxy for debug access
  - Replication controllers for tablets
  - Cloud plugins for backup/restore (Google Cloud Storage)
- More client support
  - gRPC
  - PHP
- Long-term
  - Cross-shard transactions (2PC)
  - Cross-shard joins/aggregation

Let us know what you'd like to see at vitess@googlegroups.com, or better yet...

Contribute!

- Vitess in Kubernetes on other platforms (AWS, Azure, OpenShift, ...)
    - Backup/Restore plugins (e.g. Amazon S3)
    - Startup guides (firewall rules, load balancers, etc.)
- InfluxDB (monitoring)
    - Vitess can already publish to InfluxDB, but needs work to integrate with Kubernetes
- Other client languages
    - PHP was started, but needs someone who can patch a BSON plugin for PHP

https://github.com/youtube/vitess

Questions?

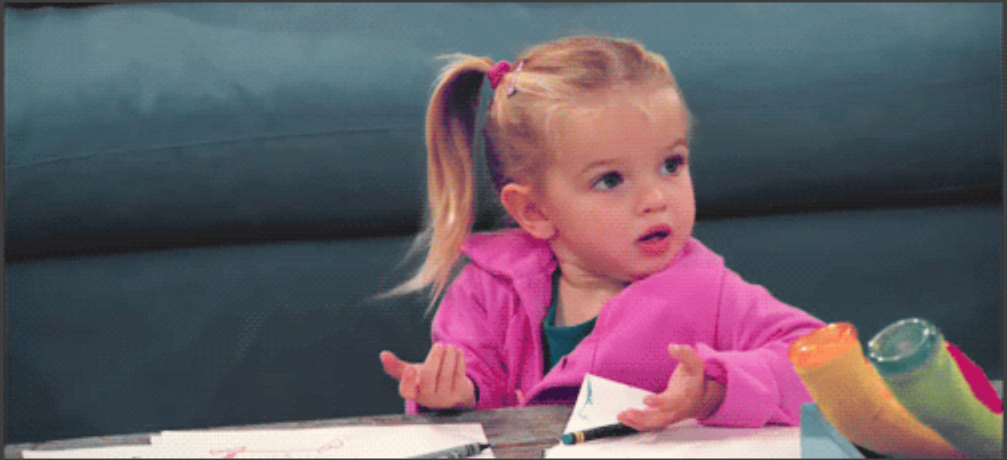

Resources

Vitess
vitess.io
github.com/youtube/vitess
vitess@googlegroups.com

Kubernetes
kubernetes.io
github.com/GoogleCloudPlatform/kubernetes

Google's MariaDB 10.0 Branch
code.google.com/p/google-mysql

Other Percona Live talks about MySQL at Google
Exploring Your Data with InnoDB Explorer - Jeremy Cole (Wednesday 1pm @ Ballroom A)
Encrypting MySQL Data at Google - Jeremy Cole, Jonas Oreland (Thursday 11am @ Ballroom D)
